# Feasibility and Safety of Flow Diversion in the Treatment of Intracranial Aneurysms *via* Transradial Approach: A Single-Arm Meta-Analysis

**DOI:** 10.3389/fneur.2022.892938

**Published:** 2022-07-15

**Authors:** Xiang Liu, Wenzhang Luo, Mingyan Wang, Changren Huang, Kunyang Bao

**Affiliations:** ^1^Department of Neurosurgery, The Affiliated Hospital of Southwest Medical University, Luzhou, China; ^2^Department of Obstetrics and Gynecology, TCM Hospital Affiliated of Southwest Medical University, Luzhou, China; ^3^Neurosurgery Clinical Medical Research Center of Sichuan Province, Luzhou, China; ^4^Academician (Expert) Workstation of Sichuan Province, The Affiliated Hospital of Southwest Medical University, Luzhou, China; ^5^Laboratory of Neurological Diseases and Brain Functions, The Affiliated Hospital of Southwest Medical University, Luzhou, China

**Keywords:** endovascular procedures, flow diversion, transradial approach, intracranial aneurysms, meta-analysis

## Abstract

**Background:**

While studies have confirmed that flow diversion (FD) can treat intracranial aneurysms *via* transradial approach (TRA), it remains unclear whether their treatment ultimately impacts safety and feasibility. We aim to conduct a systematic review and meta-analysis assessing the safety and feasibility after FD treatment of intracranial aneurysms *via* TRA.

**Methods:**

PubMed, EMBASE, and Web of Science were systematically reviewed. The primary outcomes were the success rate and the access-related complications of deploying FD *via* TRA. Meta-analysis was performed using a random or fixed effect model based on heterogeneity. And the publication bias was evaluated using a funnel plot. This study was registered with PROSPERO, number CRD42021244448.

**Results:**

Data from 8 studies met inclusion criteria (250 non-duplicated patients). The success rate was 93% (95% confidence interval [*CI*] 0.86–0.98; *I*^2^ = 61.05%; *p* = 0.01). The access-related complications rate was 1% (95% *CI* 0–0.03; *I*^2^ = 0.00%; *p* < 0.01). The mainly access-related complications included radial artery spasm (85.7%) and radial artery occlusion (14.3%). The TRA convert to transfemoral approach (TFA) was 7% (95% *CI* 0.02–0.14; *I*^2^ = 61.05%; *p* = 0.01).

**Conclusions:**

Although TFA is still the main access for FD in the treatment of intracranial aneurysms, the TRA also has a higher success rate and lower access-related complications rate. With the improvement of future experience and equipment, the TRA may become the main access for FD which has more advantages. Future studies should design prospective, multicenter randomized controlled studies for long-term follow-up.

## Introduction

In interventional cardiology, the advantages of the TRA are more and more obvious than that of the TFA (1, 2). Meanwhile, TRA gradually began to pay attention to the field of neurointervention (3–5).

With the development of Interventional Neurology, flowdiversion (FD) has become an important complementary treatment for coils and stents (6). Although some studies have shown that FD is effective and safe for the treatment of intracranial aneurysms *via* TRA, its data are limited. There were only some meta-analyses of diagnostic cerebral angiography and mechanical embolectomy (7, 8). Therefore, we conducted the first meta-analysis to illustrate the feasibility and safety of FD in the treatment of intracranial aneurysms *via* TRA. This study may be helpful to provide benchmark numbers to guide surgeons choose the appropriate access when using FD to treat intracranial aneurysms.

## Methods

This study was performed following the Preferred Reporting Items for Systematic Reviews and Meta-Analyses (PRISMA) guidelines (9).

### Search Strategy

We conducted a comprehensive literature search of the PubMed, EMBASE, and Web of Science databases for studies published from their dates of inception to May 2021. The title and abstract were searched using combinations of the following search terms: (divert OR diverts OR diversion OR flow-diverter OR flow diversion OR pipeline embolization device OR PED OR pipeline OR flow diverters OR diverters) AND (Intracranial Aneurysm OR Aneurysms, Intracranial OR Intracranial Aneurysms OR Aneurysm, Intracranial OR Brain Aneurysm OR Aneurysm, Brain OR Aneurysms, Brain OR Brain Aneurysms OR Cerebral Aneurysm OR Aneurysms, Cerebral OR Cerebral Aneurysms OR Aneurysm, Cerebral) AND (Radial Artery OR Arteries, Radial OR Artery, Radial OR Radial Arteries OR transradial OR radial OR transradial access OR transradial approach).

### Selection Criteria

The inclusion criteria were (1) treatment of aneurysms with FD *via* TRA; (2) ≥5 patients with an aneurysm; (3) and the clinical or angiographic outcomes of aneurysms reported. The exclusion criteria were as follows: (1) unextractable or unclear data; (2) duplicated reports; (3) meta-analyses, reviews, comments, letters, and non-English language studies.

### Data Extraction and Item Definition

The following information was extracted from the included studies: first author, publication year, the number of procedures treated by FD *via* TRA, baseline patient information, the number of stents successfully placed *via* the TRA, the access-related complications, and the number of the conversion from the TRA to the TFA. Data extraction was performed by Xiang Liu and Wenzhang Luo. Any disagreement during article selection was resolved by a discussion with a third author (Changren Huang). The success rate refers to the successful placement of FD *via* TRA rather than *via* TFA. The access-related complications include radial artery spasm, radial artery occlusion, forearm hematoma, and forearm osteofascial space syndrome.

### Critical Appraisal

The study quality was assessed using the modified Newcastle–Ottawa scale for case series (10). It mainly includes selection, ascertainment, causality, and reporting.

### Statistical Analysis

This meta-analysis was performed using Stata, version 14.0 (StataCorp, College Station, Texas, USA). The main outcome was the success rate of procedures and the access-related complications. The secondary result was the conversion rate of TRA. Continuous variables are presented as mean values. Dichotomous variables are presented as efficient with 95% confidence intervals (*CI*). The statistical heterogeneity was assessed using *I*^2^. A fixed-effects model was used if *I*^2^ < 50% and a random-effects model was used if *I*^2^ > 50%. An alpha level of significance was set to 0.05 and 95% *CI*.

## Results

### Search Results

**Figure 1:**
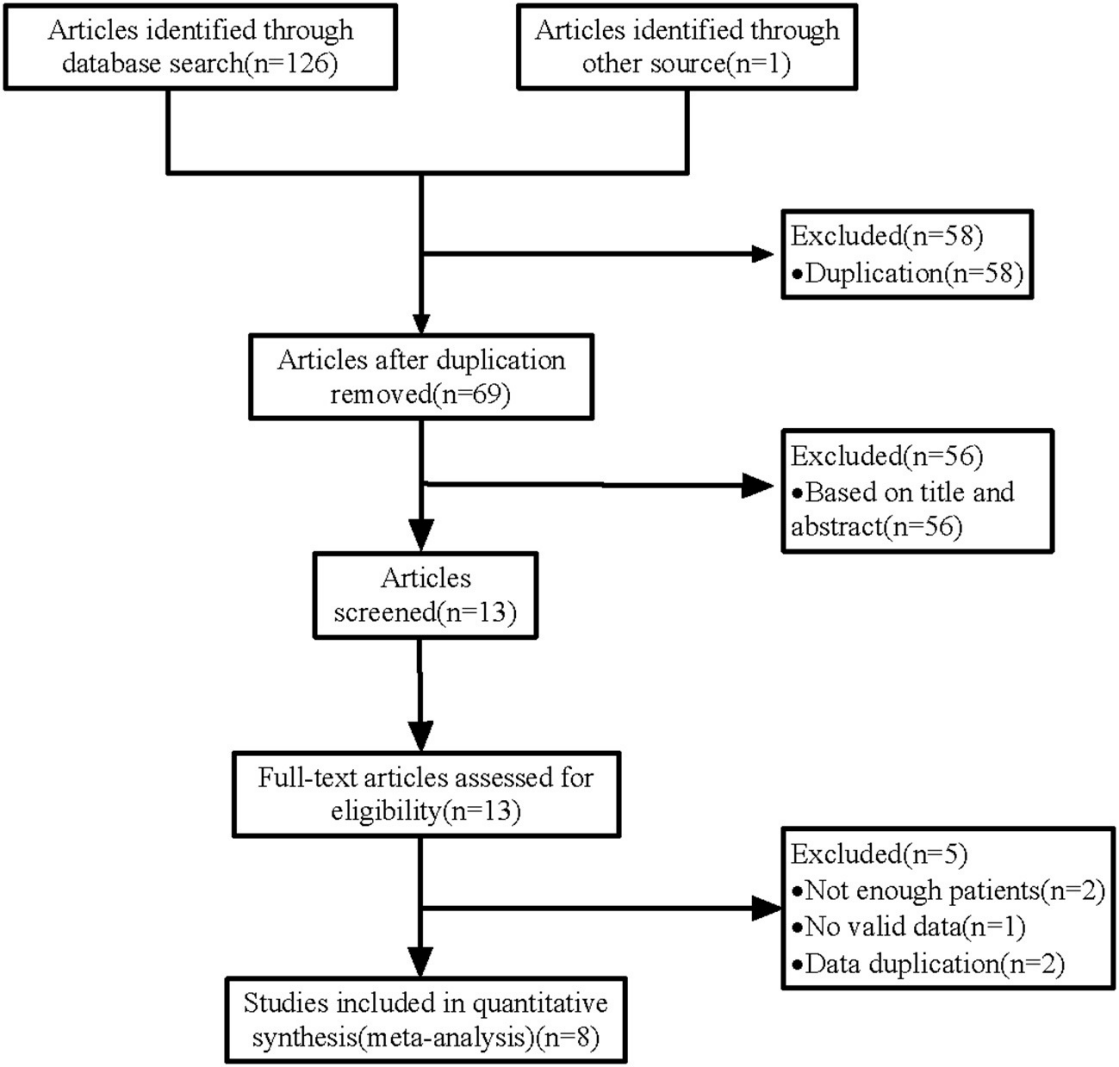
Flow chart of the study selection.

Our search rendered 127 studies (Figure 1). After duplicate removal and abstract screening, 13 studies remained for full-text screening. After reading the full text, we included 8 studies (11–18) in the meta-analysis. The 8 studies involved 265 FD-treated aneurysms *via* TRA in 250 patients. All studies were single- or multi-center retrospective analyses. Table 1 shows the quality evaluation of 8 studies and their characteristics are summarized in Table 2.

**Table 1:** Evaluation of the included studies using the criteria described by Murad et al. (10).

**Study**	**Selection**	**Ascertainment**		**Causality**		**Reporting**
	**Does the patient(s)** **represent(s) the whole** **experience of the** **investigator (center)?^a^**	**Was the exposure** **adequately** **ascertained?**	**Was the outcome** **adequately** **ascertained?^b^**	**Were other** **alternative causes** **that may explain the** **observation ruled** **out?**	**Was follow-up** **long enough for** **outcomes to** **occur?**	**Is the case(s)** **described with** **sufficient** **details?^c^**
Khandelwal et al. (12)	Yes	Yes	Yes	Yes	NR	Yes
Kühn et al. (14)	Yes	Yes	Yes	Yes	Yes	Yes
Waqas et al. (13)	Yes	Yes	Yes	Yes	Yes	Yes
Weinberg et al. (11)	Yes	Yes	Yes	Yes	Yes	Yes
Chen et al. (17)	Yes	Yes	Yes	Yes	NR	Yes
Snelling et al. (16)	No	Yes	Yes	Yes	NR	Yes
Sweid et al. (15)	Yes	Yes	Yes	Yes	Yes	Yes
Goland et al. (18)	No	Yes	Yes	No	NR	Yes

*NR means not reported.*

a *This criterion is to report only FD for intracranial aneurysms.*

b *This criterion does not meet the definition of access-related complications or there is no reason for approach conversion.*

c *This definition explains the procedure technique in detail*.

**Table 2:** The characteristics of FD in the treatment of intracranial aneurysm *via* TRA.

**Study**	**No.** **procedures**	**Mean** **Age (yr)**	**Side**	**Location**	**No. success** **(*n*%)**	**No. access-** **related** **Complication**	**Conversion** **(radial-** **femoral)**	**Reasons for** **conversion**
Khandelwal et al. (12)	29	55	R:15 L:14	AC:29	26 (90%)	3	3	Radial artery spasm 3
Kühn et al. (14)	74	57.5	NA	AC:80 PC:6	71 (96%)	1	3	vessel tortuosity 2 aberrant right subclavian artery 1
Weinberg et al. (11)	32	56.7	R:11 L:20 M:1	AC:30 PC:2	32 (100%)	0	0	0
Waqas et al. (13)	35	62.1	L:16	AC:29 PC:6	33 (94%)	0	2	vessel tortuosity 2
Chen et al. (17)	49	57.8	L:32 R:17	AC:44 PC:5	39 (80%)	2	10	LCCA angle of origin 4 LCCA/ICA tortuosity 4 Radial artery spasm 2
Snelling et al. (16)	11	NA	L:9 R:2	AC:10 PC:1	8 (73%)	1	3	Radial artery spasm 1 vessel tortuosity 2
Sweid et al. (15)	18	57.7	NA	AC:16 PC:2	17 (94%)	0	1	inadequate support 1
Goland et al. (18)	5	58.2	L:1 R:4	NA	5(100%)	0	0	0

*AC, Anterior circulation; PC, posterior circulation; LCCA, left common carotid artery; ICA, Internal carotid artery; NA, Not Available*.

### Clinical Characteristics

The average age of the 8 studies included was 55–62.1 years. Six studies (11–13, 16–18) reported the laterality of aneurysms, 57.1% (92/161) of which were located on the left side and 7 studies (11–17) reported that 91.5% (238/260) of aneurysms were located in the anterior circulation. The 8 studies described in detail the use of drugs and operative procedures before operation.

### Procedural Success

**Figure 2:**
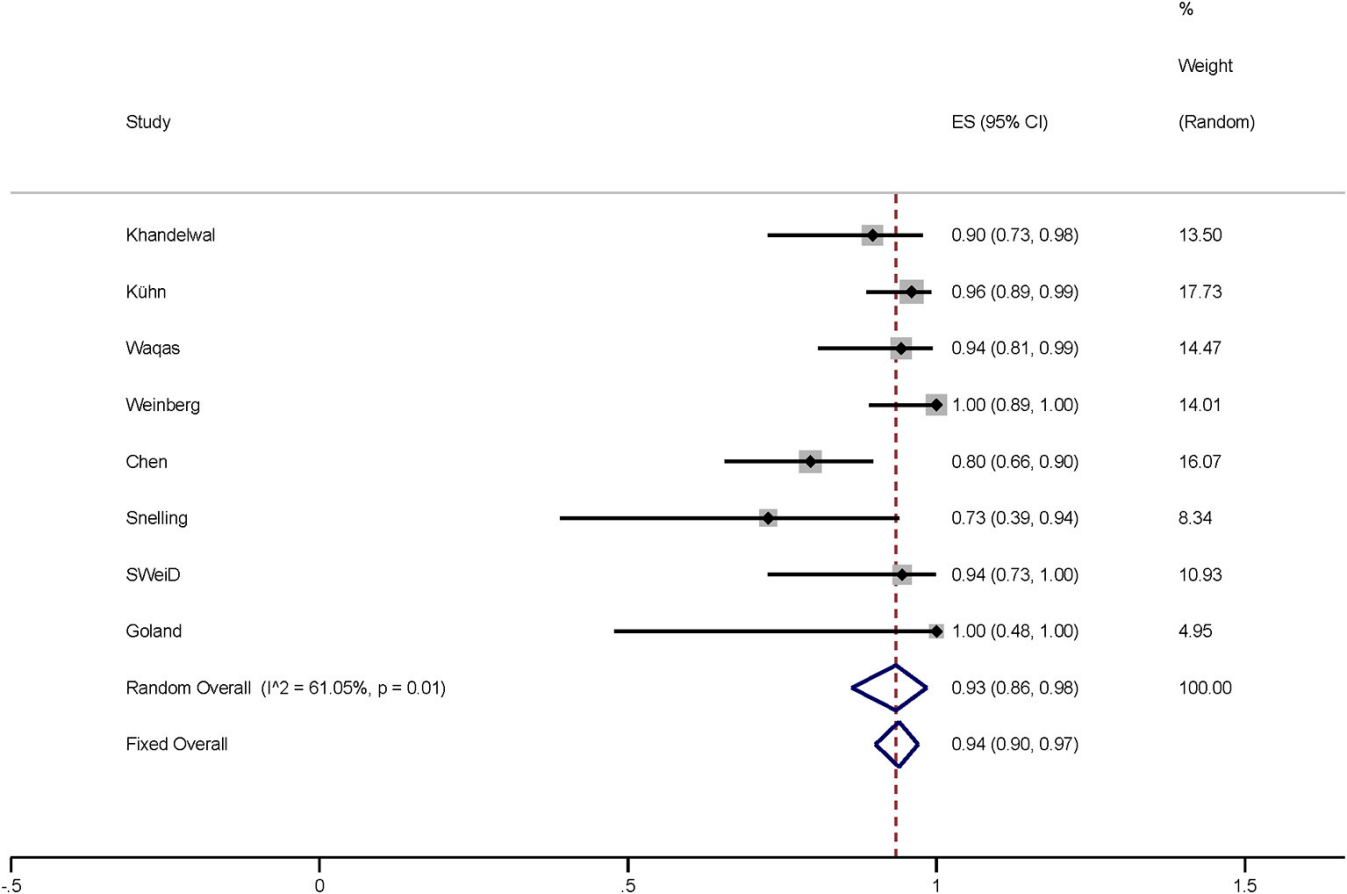
Plot showing the success rate of 265 FD-treated intracranial aneurysms *via* TRA, reported by eight studies. FD, flow diversion; TRA, transradial approach; *CI*, confidence interval.

In this meta-analysis, 253 cases of intracranial aneurysms were treated with FD *via* TRA, of which 231 cases were successful. Based on the meta-analysis of random effects, the total effect amount of 8 studies was 93% (95% *CI* 0.86–0.98; *I*^2^ = 61.05%; *p* = 0.01; Figure 2). The funnel plot showed there was no significant publication bias.

### Complications

**Figure 3:**
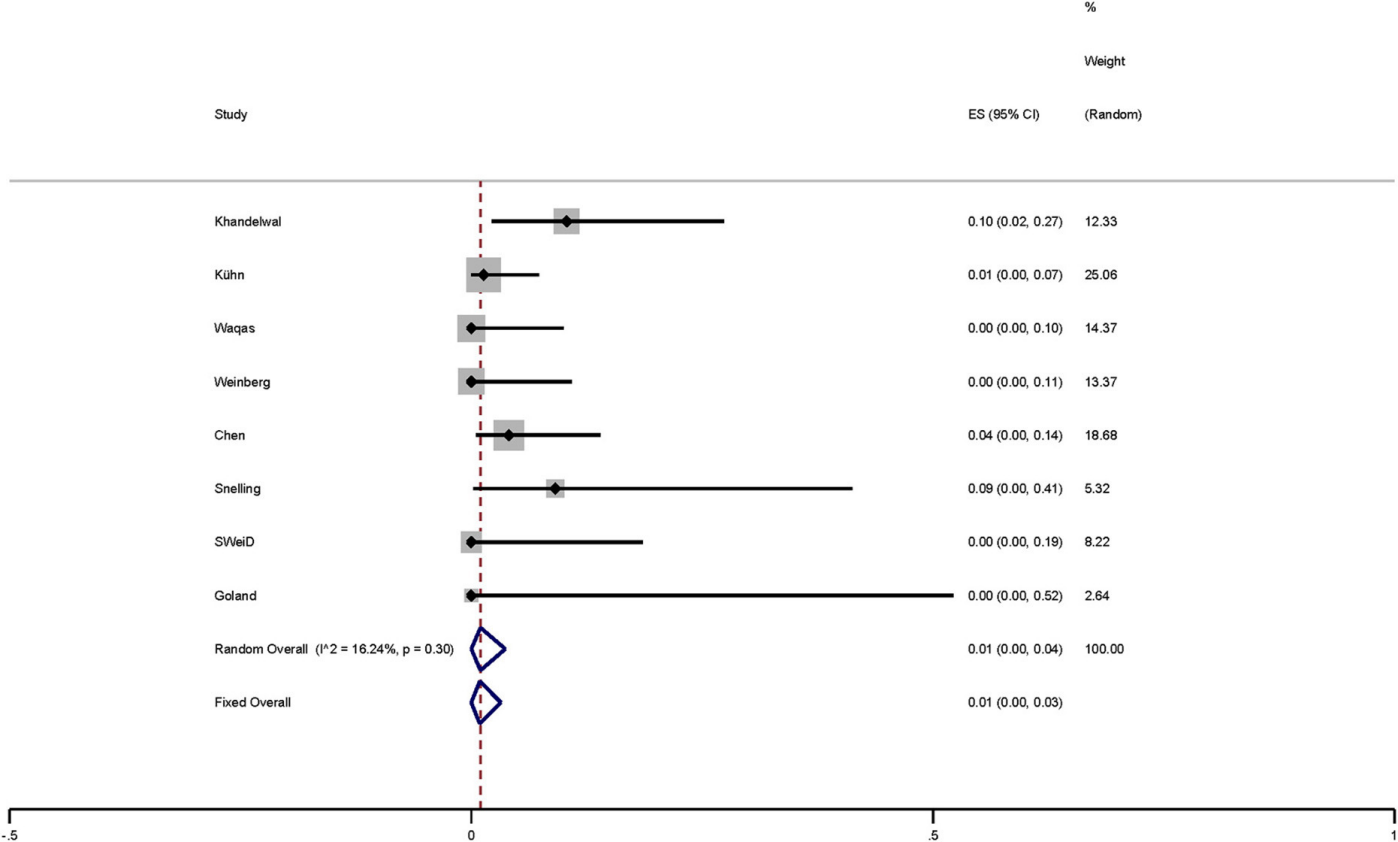
Plot showing the access-related complications of FD-treated intracranial aneurysms via TRA.

The access-related complications include radial artery spasm and radial artery occlusion. Complications occurred in four of these studies (12, 14, 16, 17). Based on the meta-analysis of fixed effects, the access-related complications rate was 1% (95% *CI* 0–0.03; *I*^2^ = 0.00%; *p* < 0.01; Figure 3). These complications included radial artery spasm (85.7%, 6/7) and radial artery occlusion (14.3%, 1/7). The funnel plot showed there was no significant publication bias.

### Conversion (Radial-Femoral)

**Figure 4:**
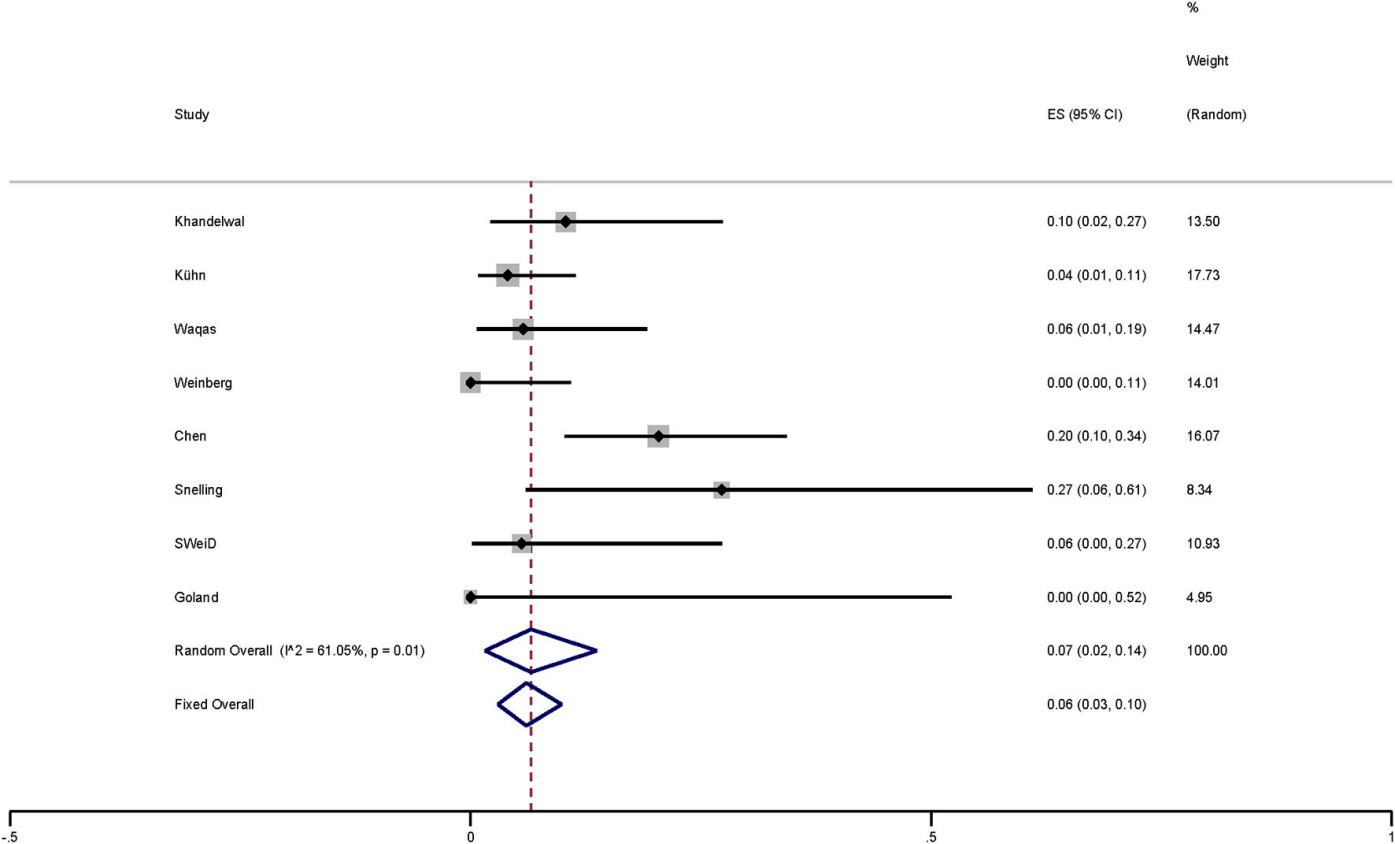
Plot showing the from TRA to TFA of FD-treated intracranial aneurysms via TRA.

Based on the meta-analysis of random effects, the conversion rate is 7% (95% *CI* 0.02–0.14; *I*^2^ = 61.05%; *p* = 0.01; Figure 4). The vessel tortuosity was the most common reason (45.5%), followed by radial artery spasm (27.3%), left common carotid artery (LCCA) angle of origin (18.2%), and inadequate support (4.5%) and aberrant right subclavian artery (4.5%). In one study (14), two cases were converted to femoral artery pathway because of vascular tortuosity and insufficient support. We think that it was caused by vascular tortuosity.

## Discussion

We present the first meta-analysis demonstrating the success rate and the access-related complications rate of FD *via* TRA for the treatment of intracranial aneurysms. Our results demonstrate that the success rate was 93% (95% *CI* 0.86–0.98; *I*^2^ = 61.05%; *p* = 0.01) and the access-related complications rate was 1% (95% *CI* 0–0.03; *I*^2^ = 0.00%; *p* < 0.01).

The concept of “endovascular flow diversion” was proposed on the assumption that the stent can block the blood flow in the aneurysm while preserving the flow into the parent vessel and adjacent branches (19). This device has higher surface coverage and lower porosity, which can slow down the blood flow to the aneurysm, gradually form thrombosis and promote the formation of new endothelium at the neck of the aneurysm (20). With the development of materials, FD has not only been confined to the original indications, but also has been applied to acutely rupture aneurysms, posterior circulation aneurysms, carotid-cavernous fistulas, distal anterior circulation aneurysms, and blister aneurysms (21, 22). At present, FD is the most commonly used access for the treatment of intracranial aneurysms *via* TFA. However, with the advantages of TRA becoming more and more prominent, some studies began to treat intracranial aneurysms with FD *via* TRA.

Dietrich et al. (23) first reported that a large cavernous internal carotid artery aneurysm was treated with Pipeline Embolization Device (PED) *via* TRA due to a complex aortic arch. Other previous studies had also reported that FD in the treatment of intracranial aneurysms *via* TRA was mainly suitable for type III aortic arch or bovine arch configurations (24, 25). At present, it has been the preferred access in some institutions with rich experience in the treatment of intracranial aneurysms with FD *via* TRA (11, 13, 15). When the radial artery is less than 2.0–2.5 mm on ultrasound, some studies suggest that TFA should be chosen. Even if you want to use TRA, 071″ systems (Envoy DA with 058″ Navien or 044″ DAC) or triaxial system should be selected (17, 23). In terms of materials, the research had shown that the system suitable for TFA is also suitable for radial artery (16). At the same time, some studies suggested Barbeau testing to evaluate palm blood circulation before operation and exclude patients with a D-shape of Barbeau testing when deploying FD *via* TRA (16, 17). However, some studies suggested that there was no additional benefit of preoperative Barbeau testing or Allen testing for hand ischemic complications (26, 27). In our meta-analysis, only 1 case had an access-related complication of asymptomatic radial artery occlusion. In addition, all researchers performed radial artery punctures under the guidance of ultrasound in the literature we included, which significantly improved the success rate of punctures. The TRA with Ultrasound Trial (RAUST) confirmed that ultrasound guidance was helpful for the success rate and efficiency of radial artery catheterization. Compared with palpation, fewer attempts for successful puncture with the guidance of ultrasound (mean: 1.65 ± 1.2 *vs*. 3.05 ± 3.4, *p* < 0.0001) (28). After a puncture, immediately 2.5–5 mg verapamil and 200 μg nitroglycerin will be paid to prevent radial artery spasm, and some studies will also be given 5 mg nicardipine (11, 15). In our meta-analysis, the incidence of radial artery spasm was 85.7% and 27.3% turned to TFA because of radial artery spasm. Therefore, how to prevent radial artery spasm is also one of the problems that TRA will become the main access for the treatment of aneurysms with FD in the future. There are also reports of forearm hematoma and forearm osteofascial space syndrome during interventional operation *via* TRA (29, 30). A short sheath of 6F was routinely inserted after the puncture, and the target artery was entered through Simmons-II. An appropriate multiaxial system was supposed to place FD according to the diameter of the radial artery. Because the deployment of FD requires a larger vessel diameter, it not only increases the risk of radial artery spasm but also makes surgeons reluctant to deploy FD *via* TRA. The triaxial system was utilized for patients with radial artery diameter > 2.5 mm and the biaxial or triaxial system was used for patients with radial artery diameter <2.5 mm in a multicenter study. The overall success rate was 91% (122/134) and compared with TFA, which has higher access-related complications (2.48 *vs*. 0%, *p* = 0.039) (30). Their research also believed that the deployment of FD *via* TRA is safe and feasible. Patel et al. (31) believed that the biaxial systems could replace the triaxial systems to place FD. In our meta-analysis, the incidence of access-related complications is only 1% (95% *CI* 0–0.03; *I*^2^ = 0.00%; *p* < 0.01). On the other hand, the most important reason is that the access conversion is 63.7% owing to the vessel tortuosity and LCCA angle of origin. In the future, the development of neurointervention materials and the progress of technology may improve this situation.

Deploying FD needs to take a large dose of dual antiplatelet therapy, which increases the risk of femoral artery bleeding, prolonged compression time, and pseudoaneurysm *via* TFA (32). The radial artery is shallow, which is easier to stop bleeding by compression. At the same time, the TRA will not lead to the patient's bed for a long time, and will also reduce the incidence of lower extremity deep venous thrombosis (15, 33, 34). Secondly, several studies had shown that neurointerventional *via* TRA can reduce the discomfort of patients after interventional surgery, and reduce the cost of surgery, and length of hospital stay compared to TFA (3, 11, 35–37). Especially for patients taking anticoagulants, pregnant women, patients with severe iliac atherosclerosis, bovine arch, type II/III aortic arch, the TRA should be the main access for FD in the treatment of intracranial aneurysms (11, 38, 39).

Although the deployment of FD *via* TRA has more benefits for patients with intracranial aneurysms, it also has a high success rate in our meta-analysis. However, we cannot ignore the causes of his conversion to TFA and its complications. We should choose the appropriate access based on maximizing the interests of patients.

### Limitations

Our study has some limitations. We only included a small number of cases without a control group which lead to selection bias, and this result is not suitable for comprehensive promotion. Further, we were unable to analyze the location of the failed aneurysm, aortic arch angles, and catheter system because of the lack of stratification. Moreover, given the lack of long-term follow-up in the included studies, we were not able to consider the cases of access-related complications that may have been missed.

## Conclusions

Although TFA is still the main access for FD in the treatment of intracranial aneurysms, the TRA also has a higher success rate and lower access-related complications rate. With the improvement of future experience and equipment, the TRA may become the main access for FD which has more advantages. Future studies should design prospective, multicenter randomized controlled studies for long-term follow-up.

## Data Availability Statement

The original contributions presented in the study are included in the article/supplementary material, further inquiries can be directed to the corresponding author.

## Author Contributions

Guarantor of integrity of entire study, manuscript revision and review, manuscript final version approval, and manuscript definition of intellectual content: KB and CH. Study concepts: XL and MW. Study design and data acquisition: XL and WL. Literature research: MW. Data analysis/interpretation and statistical analysis: XL, WL, and KB. Manuscript preparation: XL. All authors contributed to the article and approved the submitted version.

## Funding

This study was funded by the Scientific Research Project of Sichuan Provincial Health Committee, 19PJ296; Luzhou Science and Technology Plan Project, 2020-SYF-29.

## Conflict of Interest

The authors declare that the research was conducted in the absence of any commercial or financial relationships that could be construed as a potential conflict of interest.

## Publisher's Note

All claims expressed in this article are solely those of the authors and do not necessarily represent those of their affiliated organizations, or those of the publisher, the editors and the reviewers. Any product that may be evaluated in this article, or claim that may be made by its manufacturer, is not guaranteed or endorsed by the publisher.

## Abbreviations

FD: flow diversion
CI: confidence interval
TRA: transradial approach
TFA: transfemoral approach
LCCA: left common carotid artery
AC: anterior circulation
PC: posterior circulation
ICA: internal carotid artery
NA: not available.
